# Editor’s Outlook

**Published:** 1893-06

**Authors:** 


### EDITOR'S OUTLOOK.

### SUMMER RECREATIONS.

In order to derive the highest advantages as to health, from summer recreations, several considerations ought to be kept in view.

Children who are teething should be taken without an hour's delay to the sea shore. The effect is, in multitudes of cases, instantaneous, radical and almost miraculous. Physicians of observation in large cities will testify that children in their second summer, in an almost dying condition begin to improve in their journey to the coast, and within three hours after leaving the heated and sultry atmosphere of the city in midsummer.

There is something in the salt air of the sea which has a renovating and life giving power to all whose brains have been overtaxed; and to many whose nervous symptoms have been impaired by intense excitements, whether arising from business anxieties, or domestic calamities.

There is also a moral effect for good in the roar of the ocean and in the sense of vastness which comes over the mind as the eye gazes upon it, bottomless and without a shore beyond, thus causing heart troubles to be swept away in their significance. To merchants, clerks, lawyers, to all who follow sedentary occupations, who are kept within four walls for a large portion of every twenty-four hours, no better advice can be given than to go off among the mountains, climb to their tops, descend into their valleys, penetrate their recesses, on foot, on horse, in every conceivable mode of locomotion, and they should consider every hour of daylight lost which does not find them in interested motion in the open air. The general rule is to effect a change of air.

Any change is more or less beneficial. There is no locality in any dozen miles apart whose atmosphere has not ingredients differing in some respects from that of other localities, and the human system readily drinks in those new or strange ingredients, just as one takes in, with unwonted delight and benefit, the food of a table a few miles from his own home. Both mind and body the world over yearn for variety, for change. So that a man living for years in the purest atmosphere on earth will be benefited by a change to one which although relatively less pure, has either different ingredients or the same in different proportions. To all who can, we say go somewhere, go anywhere rather than remain at home all the time. Go with as light a heart as possible ; go determined to get good and do good, and you will seldom fail of both.

But in going leave all "airs" and mocks and pretenses and shams behind.

Assume nothing ; exact nothing ; claim nothing beyond what is spontaneously offered by those with whom you may come in contact. In all situations be courteous, and respect yourself and you will have respect and courtesy shown you. Acting thus you will return home healthier, happier, wiser and better than when you went away.

---

To give all our readers plenty of opportunity to secure the Chambers Encyclopedia, we have decided to hold open our offer until September first, when it will then close.

The publishers are gratified at the many expressions of satisfaction and kind words already received from our offer, and consequently we feel that our efforts are appreciated. We desire to impress upon all that by offering such a handsome premium it will not depreciate in the least the high standard that the JOURNAL has attained in its forty years of existence. On the contrary we shall make improvements from month to month, and will always be in keeping with the times.

The publishers take the liberty to publish a testimonial from one of our recent subscribers :

SALEM, Mass., April 20.

Publishers HALL'S JOURNAL OF HEALTH :

Your Encyclopedia and the JOURNAL for May, came to hand promptly, and I am delighted with them. The Encyclopedia is a valuable acquisition to our household, and must say, again that I am more than gratified and pleased.

May thousands accept of your efforts to place such a treasury of knowledge before them, and with best wishes, I am yours sincerely,

10 Mason Street. B. W. NASON.

---

Our issue for July will appear much earlier than usual on account of our Recreation Bureau.

We are very much gratified at the results of our attempt to make this department a special feature of the JOURNAL.

Daily inquiries are passed through the Bureau, and we again invite all who desire information about any resort in the world, knowledge of its hotels, surroundings, etc., how to reach them, the cost, etc., to address the Recreation Bureau, care of the JOURNAL, and circulars and information will be promptly sent.

In the next issue we propose to publish a guide to all the prominent summer resorts, and it is especially requested that hotels who desire representation in it to forward their circulars *at once.*

